# Consumption of Ultra-Processed Food and Its Association with Sociodemographic Characteristics and Diet Quality in a Representative Sample of French Adults

**DOI:** 10.3390/nu13020682

**Published:** 2021-02-20

**Authors:** Giovanna Calixto Andrade, Chantal Julia, Valérie Deschamps, Bernard Srour, Serge Hercberg, Emmanuelle Kesse-Guyot, Benjamin Allès, Eloi Chazelas, Mélanie Deschasaux, Mathilde Touvier, Carlos Augusto Monteiro, Renata Bertazzi Levy

**Affiliations:** 1 Department of Preventive Medicine, School of Medicine, University of São Paulo (FMUSP), São Paulo 01246-903, Brazil; rlevy@usp.br; 2 Center for Epidemiological Studies in Health and Nutrition, University of São Paulo (Nupens/USP), São Paulo 01246-904, Brazil; carlosam@usp.br; 3 Sorbonne Paris Nord University, Inserm, INRAE, Cnam, Nutritional Epidemiology Research Team (EREN), Epidemiology and Statistics Research Center–University of Paris (CRESS), 93017 Bobigny, France; julia@eren.smbh.univ-paris13.fr (C.J.); valerie.deschamps@univ-paris13.fr (V.D.); b.srour@eren.smbh.univ-paris13.fr (B.S.); s.hercberg@uren.smbh.univ-paris13.fr (S.H.); e.kesse@eren.smbh.univ-paris13.fr (E.K.-G.); b.alles@eren.smbh.univ-paris13.fr (B.A.); e.chazelas@eren.smbh.univ-paris13.fr (E.C.); m.deschasaux@eren.smbh.univ-paris13.fr (M.D.); m.touvier@eren.smbh.univ-paris13.fr (M.T.); 4 Public Health Department, Avicenne Hospital, AP-HP, 93017 Bobigny, France; 5 Santé Publique France, The French Public Health Agency, Nutritional Epidemiology Surveillance Team (ESEN), 93017 Bobigny, France; 6 Department of Nutrition, School of Public Health, University of São Paulo (FSP/USP), São Paulo 01246-904, Brazil

**Keywords:** food processing, ultra-processed food, diet quality, France

## Abstract

The present study aims to describe ultra-processed food (UPF) consumption in a representative sample of French adults and to evaluate the association between UPF consumption and socioeconomic characteristics and nutritional profile of the diet. This is a cross-sectional study using food consumption data from the Étude Nationale Nutrition Santé (ENNS), conducted with 2642 participants (18–74 years old), between February 2006 and March 2007 in France. Dietary data were collected through three 24-h dietary recalls. All food and beverages were classified according to the NOVA classification. The energy contribution of NOVA food groups to total energy intake was presented by categories of sociodemographic characteristics. Linear and logistic regression models were used to estimate the association between the percentage of UPF in the diet with nutritional indicators. The mean daily energy consumption of the adult French population was 2111 kcal, of which 31.1% came from UPF. This percentage was higher among younger individuals, and in the urban area, and lower among individuals with incomplete high school and individuals who were retired. The consumption of UPF was positively associated with the dietary energy density and the dietary contents of total carbohydrates, free sugar, and total and saturated fat, as well as with inadequate dietary energy density, saturated fat, free sugar, and fiber intakes.

## 1. Introduction

France is recognized worldwide for its traditional culinary practices. The techniques of food transformation and culinary techniques developed over the years have highlighted France for the manufacture of refined products and also for its exquisite cuisine, characterizing the country for its strong food culture [1,2]. Subsequently, studies have pointed out the possible health benefits provided by traditional French eating habits and in particular the Mediterranean diet, considered a protective factors against the development of cardiovascular diseases and all-cause mortality [3,4,5,6,7].

Even though the traditional food culture is still predominant in France, changes in food consumption have been observed. Studies conducted periodically with a representative sample of the French population (Étude Individuelle Nationale des Consommations Alimentaires (INCA) 1 and 2) have shown changes in food consumption among adults, highlighting a decrease in the consumption of traditional foods, such as milk, cheese, meat, bread, potatoes, homemade pastries/puff-pastries, and sugar/confectionery [8]. In its most recent version (INCA 3), the study noted an increased share of industrially processed foods in the diet [9].

Changes in dietary patterns is one of the factors that has affected the French population’s nutritional status, and possibly contributed to the increase in the prevalence of obesity in children and adults, though the prevalence of obesity in the country remains low compared to its European counterparts [10]. Between 1997 and 2006, the prevalence of obesity in the French population increased from 8.6% to 13.1% [10]. Between 2006 and 2015, the increase in obesity was not statistically significant, though the prevalence remained somewhat high, reaching 16.8% of men and 17.4% of women aged 18 or older [10].

The convenience of industrially processed foods compared to unprocessed and minimally processed foods coupled with the strong advertising, the vast food supply, and the high dynamism and innovation of the food industry are factors that may partly explain changes in diet [11].

The association between food processing and diet quality and health of the French population has been evaluated through a series of studies that used the NOVA (non-acronym name) classification and data from the NutriNet-Santé cohort [12,13,14,15,16,17,18,19,20]. According to NOVA, ultra-processed foods (UPFs) are the formulation of food substances involving processes and ingredients exclusively employed in industrial production [21,22,23].

Although studies conducted with the NutriNet-Santé cohort provide valuable information on UPF consumption in the French population and its association with health outcomes, using data from a representative national sample with rigorous sample strategy is paramount to accurately estimate the extent of UPF consumption in the general population.

In this context, the present study aims to describe dietary intake according to food processing degree as well as the association between UPF consumption and nutritional profile of the diet in a representative sample of the French adult population.

## 2. Materials and Methods

### 2.1. Design and Population

This is a cross-sectional study based on individual food consumption data from the Étude Nationale Nutrition Santé (ENNS) survey, conducted by the French Public Health Agency (Santé Publique France) between February 2006 and March 2007 across all territories of metropolitan France.

The study sampling strategy was determined based on the French census (for mainland France, excluding Corsica Island and overseas territories). Geographic zones were randomly selected and subsequently stratified into eight large regions according to urbanization level. The number of individuals included in each stratum was defined proportionally to the population size, with a minimum of twenty-four dwellings to be investigated. The survey was conducted in randomly selected households from telephone listings, and it included individuals between 3 and 74 years old residing in metropolitan France. Individuals residing in mobile homes, nursing homes, student housing, or detention centers, as well as people with artificial feeding (enteral or parenteral), were not included in the survey.

In the present study, only adult respondents that were aged ≥18 years old were considered. Among the 3115 adults who answered the food questionnaire, 361 individuals were excluded for under-reported diet, 108 individuals were excluded for not filling out three 24-h food records, and three individuals were excluded for not responding or refusing to answer information about schooling. The final sample size included 2642 adults aged 18 years or older.

Approval for the study was obtained from the French data protection authority (Commission nationale de l’informatique et des libertés, authorization no. 905,481) and a bioethics committee (Hôpital Cochin, Paris, no. 2264).

### 2.2. Data Collection

Sociodemographic data (marital status, number of children per family, country of birth, employment status, type of job, hours of work, education, type of housing, living standards, food insecurity, household income, and number of residents per household) were also collected for all individuals. Food consumption was evaluated using dietary recalls.

### 2.3. Food Consumption

Information about food consumption was collected using three 24-h dietary recalls. The dietary recalls were applied on weekdays and weekend days in order to evenly represent all days of the week. Additionally, the field work was conducted throughout the year in order to take into account potential seasonal variations in food consumption. The phone interviews were conducted by previously trained nutritionists, and the respondents were asked to describe as precisely as possible the amount of food and beverage consumed during the previous day, aided by a validated photographic manual of portion sizes for the French population [24]. Information on food composition, recipes (especially for homemade food), and food brands was also collected. Information on the addition of added fats as well as salt during food preparation and consumption was recorded separately. The interviews were conducted by telephone. Individuals were categorized according to age group and sex, and then participants with extremely energy intake were identify using the method proposed by Black [25]. Subsequently, cases of excessive or insufficient caloric intake without valid justification were excluded [26].

Energy and nutrient contents for all reported foods and drinks were estimated according to the national food composition table [27]. Free sugar content was estimated following the recommended method of the Pan-American Health Organization (PAHO) document “Pan American Health Organization Nutrient Profile Model” [28].

### 2.4. NOVA Classification

Food items were classified according to the extent and purpose of food processing, in accordance with NOVA. The NOVA classification divides foods into the following groups: unprocessed and minimally processed food, processed culinary ingredients, processed foods, and ultra-processed foods [21,22,23].

The unprocessed and minimally processed foods group (group 1) includes unaltered or minimally processed food (altered by processes such as removal of inedible parts, fractioning, freezing, and packaging) obtained directly from plants or animals. This group contains foods such as fruits, leafy and root vegetables, grains, legumes, meat, poultry, fish, eggs, and milk.

The processed culinary ingredients group (group 2) includes substances obtained directly from food or from nature and commonly used in culinary preparations and rarely consumed in the absence of food from group 1. This group encompasses ingredients like salt, sugar, and oils.

Processed foods are products made by adding sugar, oil, salt, or other group 2 substances to group 1 foods. Most processed foods have two or three ingredients and are either industrially prepared, artisanal, or home-made through food processing operations, such as preservation, and, for bread and cheese, non-alcoholic fermentation.

Ultra-processed food (UPF), the primarily focus of this study, refers to products that undergo industrial processes that include, for instance, hydrogenation, hydrolysis, extruding, molding, reshaping, and pre-processing by frying. Flavoring agents, colors, emulsifiers, humectants, non-sugar sweeteners, and other cosmetic additives are often added to these products to imitate sensorial properties of unprocessed or minimally processed foods and culinary preparations.

In this study, unprocessed and minimally processed foods were grouped with processed culinary ingredients, such that all foods and beverages were divided into the following three groups: unprocessed and minimally processed food and culinary ingredients, processed foods, and ultra-processed foods.

To categorize foods according to the NOVA classification, food items were categorized into their most usual form of consumption (e.g., sweetened beverages, Bolognese sauce, or cookies as ultra-processed foods; rice as minimally processed, etc.), with the most conservative classification option always chosen in case of doubt. Elements in the descriptor of the food (e.g., “fruit in syrup”) were used to help with the classification of foods. In case of uncertainty, consensus was reached among the researchers. As culinary preparations were not broken down into underlying ingredients, a subgroup named “mixed dishes” was created within minimally processed foods to include dishes frequently composed of food items from different NOVA subgroups. A reduced number of these mixed dishes presented processed or ultra-processed food items in their composition, such as ultra-processed sauces, which were not separated from the rest of the recipe.

### 2.5. Statistical Analysis

The average values of the three 24-h dietary recalls were used to describe the diet of the population. Average absolute and relative calorie intake of NOVA groups and subgroups were estimated. The absolute and relative gram contribution of food groups and subgroups was also estimated to better assess the contribution of ultra-processed, low energy density foods, such as artificially sweetened beverages.

The caloric contribution of NOVA’s food groups was described by sociodemographic characteristics. The sociodemographic variables included in the present study were as follows: sex (male and female), age group (18–39, 40–59, and ≥60 years old), living area (urban or rural), occupational category (management/intermediate profession; self-employed/farmers; manual workers/employees; retired; and homemakers, disabled persons, and others), and educational level (incomplete high school, complete high school, technical course, and university graduate).

To evaluate the nutrient intake associated with the development of non-communicable diseases NCDs [29,30,31], total energy (kcal), energy density (kcal/g), percentage of protein (% of total energy), percentage of carbohydrates (% of total energy), percentage of total sugar (% of total energy), percentage of free sugar (% of total energy), percentage of total fat (% of total energy), percentage of saturated fat (% of total energy), contents of fiber (g/1000 kcal), contents of potassium (mg/1000 kcal), and contents of sodium (mg/1000 kcal) were explored.

The contribution of each NOVA group to macro and micronutrient dietary intakes was computed. The nutrient intake was described for each diet fraction, and the confidence interval of 95% was used to assess the statistical difference between the means of each indicator.

To verify the association between UPF consumption and food consumption and nutrient intakes, first, the sample was divided according to quintiles of the UPF contribution to the diet (percentage of energy intake), with the lowest consumers belonging to the first quintile and the highest consumers belonging to the fifth quintile. Then, we estimated the overall population’s average nutrient intakes adjusted for sociodemographic characteristics for the overall diet and across quintiles of the dietary energy share of ultra-processed foods. Linear regression models were performed to evaluate the association between UPF quintiles (exposure variable) and nutrient intakes (outcome variables). To compare the coefficients across variables with different units, we used standardized regression models.

The nutrient intakes were also evaluated using the prevalence of inadequate nutrient intakes. The criteria used to estimate the prevalence of inadequate nutrients intakes were the specific recommendations for the French population, determined by the Agence nationale de sécurité sanitaire de l’alimentation, de l’environnement et du travail (Anses), which considers the following nutritional intake parameters as inadequate: fat intakes greater than 12% of the total daily calories, free sugar intakes larger than 100 g/day, and fiber intakes lower than 30 g/day. When French recommendations were not available, the World Health Organization’s nutritional recommendations (sodium intake > 1 g/1000 kcal and potassium intake < 1755 mg/1000 kcal are considered inadequate) and the criteria established by the World Cancer Research Fund (energy density < 1.25 or > 1.45 kcal/g is considered inadequate) were used. The prevalence of inadequate nutrient intakes was described for overall population and according to quintiles of UPF consumption. Odds ratios from logistic regression models were used to assess the magnitude of the associations between quintiles of energy contribution of UPF (exposure variable) and inadequate nutrient intakes (outcome variables).

Linear and logistic regression analyses were adjusted for the following potential confounders: sex, age group, living area, region, occupation, and educational level. To evaluate dose–response associations, crude and adjusted tests of linear trend were performed by treating quintiles of ultra-processed food consumption as an ordinal variable. Statistical analyses were carried out with the software Stata, version 14.1 (Statistical Analysis System, StataCorp, College Station, Texas, USA), accounting for the survey weighting factors and sample complexity.

## 3. Results

Table 1 describes the sample distribution used in the study according to sociodemographic characteristics. The final sample consisted of 2642 individuals. The majority of the participants were female (63.3%), aged between 40 and 59 years (44.8%), living in an urban area (76.9%), manual workers/employers (30.4%), and had completed high school (50.5%).

The average energy intake of French adults older than 18 years was 2110.7 kcal (CI 95% 2073.9–2147.4), from which 44.0% originated from unprocessed or minimally processed foods and culinary ingredients, 24.9% from processed foods, and 31.1% from UPFs. Within the first group, the subgroups with the largest percentage intake contributions were meat (9.0%), added fat (5.3%), and fruits (4.2%). Among processed foods, the subgroups with the largest percentage intake contributions were bread (13.5%) and cheese (6.1%). Within UPFs, the subgroups contributing the most to energy intake were ready-to-eat meals (7.9%) and confectionery (5.4%) (Table 2).

The contribution of food groups and subgroups was also evaluated according to the percentage of the total diet in grams (Supplementary Material: Table S1). When evaluated by grams, the contribution of subgroups with low energy density, such as fruits, vegetables, and beverages, increased. The contribution of minimally processed foods and culinary ingredients was 59.9% of the total diet, processed foods contributed 16.0% and UPF contributed 24.1% of the total diet. Within minimally processed food and culinary ingredients, the subgroups with the largest contributions were coffee and tea (14.6%), fruits (8.6%), and vegetables (8.2%). Among the processed foods, the subgroups with the largest contributions were beer and wine (5.6%) and bread (5.5%). Within UPFs, the subgroups contributing the most to weight intake were ready-to-eat meals (6.7%) and sweetened beverages (6.5%).

Table 3 shows the mean percentage of total energy intake from UPF according to sociodemographic characteristics. A larger contribution of UPFs to the diet was found in individuals aged between 18 and 39 years (39.1%) and urban area residents (31.9%), while individuals with incomplete high school (26.5%) and individuals who were retired presented the lowest percentages (22.3%).

The nutrient profile and fractions of nutrient intakes from unprocessed or minimally processed foods and culinary ingredients, processed foods, or UPF are presented in Table 4. The fraction of unprocessed or minimally processed foods and culinary ingredients showed the lowest energy density, and carbohydrate and sodium content as well as the highest content of protein and fiber. When compared to the other fractions, that comprising processed foods showed the highest amounts of carbohydrates and sodium as well as the lowest contents of total and free sugars, total and saturated fats. Finally, UPF fraction showed the highest contents of total and free sugars and total and saturated fats, but the lowest amounts of protein and fiber.

Table 5 shows the mean nutrient intake of the overall population and across quintiles based on the proportion of UPF in the diet, as well as the standardized and non-standardized linear trend regression coefficients between variables. Following adjustment for possible confounding factors, the quintiles in UPF consumption showed positive associations with energy density, percentage of carbohydrates, free sugar, total fat, and saturated fat, and were inversely associated with percentage of protein, as well as fiber and potassium densities. Free sugar stands out among the nutrients with the greatest association with UPF consumption—a mean increase of 1.27% was observed for the consumption of free sugars from one quintile to the next.

Table 6 shows the prevalence of inadequate nutrient intake for the whole population and across quintiles of UPF consumption strata, as well as the adjusted prevalence ratios of the regression models. The odds of inadequate nutrient intake was high among the population. About 94.7% of French adults showed inadequate sodium consumption, 93.5% showed inadequate fiber intake, 86.3% showed inadequate saturated fat intake, 76.2% showed inadequate energy density, and 76.5% showed inadequate potassium intake. The adjusted coefficients showed that UPF quintiles were positively associated with inadequate intakes of energy density, saturated fat, free sugar, fibers, and potassium, and negatively associated with inadequate sodium intake. Compared with the first quintile of UPF participation, the fifth quintile had a 6.7 higher chance of presenting inadequate consumption of free sugars, a 5.9 higher chance of presenting inadequate consumption of fibers, a 3.5 higher chance of presenting inadequate consumption of potassium, a 2.5 higher chance of presenting inadequate consumption of saturated fat, and a 2.0 higher chance of presenting inadequate consumption of energy density.

## 4. Discussion

UPFs contributed to almost one-third of the energy intake of French adults. The consumption of UPF was associated with a lower nutritional profile of the diet, namely increased energy density, carbohydrates, free sugar, total fat, and saturated fat, as well as decreased fiber and potassium intakes. Furthermore, UPF consumption was associated with a higher prevalence of inadequate intake dietary energy density and inadequate intakes of saturated fat, free sugar, fiber, and potassium.

The dietary contribution of UPF to total energy intake in France is lower than in other countries, such as the USA (57.5%), Canada (47.7%), Australia (42.0%), and the UK (56.8%) [32,33,34,35]. It is similar to the consumption levels in European countries, such as Belgium (29.6%) [36], and middle-income countries, such as Brazil (21.5%), Mexico (29.8%), and Chile (28.6%) [37,38,39].

While in developing countries the smaller percentage of UPF consumption is possibly reflecting delayed economic growth, the lower consumption in France is likely related to the traditional culinary culture. Although industrialization of the food system caused a shift in the population’s diet, evidence suggests that the traditional culinary culture still resists the “Westernization” of traditions, probably due the great appreciation of the French “haute cuisine” and also the “rustic/regional cuisine” [1].

In France, UPF consumption showed an inverse relationship with age as well as a direct association with residence in urban regions, findings that were previously observed both in developed and developing countries [35,36,39,40]. The high UPF consumption observed among the youngest individuals is presumably caused by their ability to easily accept new eating habits and innovations [9] as well as intensive marketing of manufactured products directed toward young citizens [41].

The magnitude of UPF consumption and the distribution of food items within this group vary between countries, but in all countries, the intake of UPFs was associated with poorer nutritional profiles of diet. Previous studies conducted in the United States, Canada, the UK, Belgium, Australia, Brazil, Chile, and Colombia found negative associations between UPF consumption and the intake of nutrients known to protect against NCDs and positive associations with negative diet markers [32,33,34,36,37,38,39,42,43,44,45,46] and with the prevalence of inadequate nutrient intake, which is linked to the development of NCDs [33,34,39]. The present study presented similar results. Although the magnitude of some coefficients was low, the indicators go in the same direction, indicating that the high consumption of ultra-processed products negatively impacts several indicators in the diet. So, together, the measures show that a diet rich in ultra-processed products has a low nutritional profile compared to a diet predominated by non-ultra-processed foods.

The UPF consumption in the present study showed an inverse association with inadequate sodium intake, suggesting that people eating a lower quantity of UPF had higher sodium intake. These findings might be explained by the high content of sodium in traditional French cuisine, such as artisanal cheese and bread (the main contributor to sodium intake in France [47], which belonged to the NOVA 3 group (“processed”) and not the NOVA 4 (UPF) group.

A study conducted in France within the NutriNet-Santé cohort showed a positive association between sodium intake and the percentage of UPF in the diet, contrary to what was found here [12]. These discrepancies may be related to differences in the populations included in the studies.

The present study had limitations. Although it is a nationally representative study, individuals in situations of vulnerability (such as citizens living in nursing homes and individuals without a telephone line) were not well captured by the study. Data were collected between 2006 and 2007, and may not represent current food consumption patterns, however, new studies will address this limitation.

Specific food subgroups, such as candies, sweets, and snacks, are often underreported in dietary surveys due to desirability bias and this may underestimate the consumption of ultra-processed foods. Additionally, data found in the food consumption module of the survey do not always contain enough information to categorize food according to the NOVA classifications, since the sampling instrument was not developed with the aim to classify foods according to the degree of industrial processing. When present, information about the brand of the foods helped in the categorization, but when it was not possible to distinguish culinary preparations and industrialized food, the most conservative categorization was chosen (the one with the lower degree of processing). Thus, this may have led to an underestimation of the UPF consumption and attenuated the associations found.

Analyses to control inter- and intra-variability were not performed, and although several 24-h records were used and weighted according to weekend and weekdays, they do not fully capture all intra-variability in dietary intakes, and caution is needed in the extrapolation of these estimates to measure the “usual diet”. However, any error related to diet variability would be mitigated, since the average of all days of intake in the dietary assessment of the exposure and the outcome were used, therefore minimizing the effect on the study conclusions.

Considering mounting scientific literature linking UPF to adverse health outcomes [13,14,15,16,17,18,19,20], an increasing number of countries have started to implement public policies to limit their consumption. Approaches such as taxes, restricting where these products can be sold, and advertisement regulations have been utilized in some countries [48,49]. In that context, laws implemented in France are promising ways to regulate the consumption of highly processed food. For example, beverages sweetened with sugar and sweeteners started to be overtaxed in January of 2012, by the article 1613 of the Code général des impôts [50]. Such policies could be considered for expansion to include other ultra-processed products, thereby discouraging their consumption.

Concomitantly with measures restricting UPF sales, it is also important to implement educational actions to raise awareness among the population about the potential harm and risks associated with consuming these products. In France, the Programme National Nutrition Santé (PNNS) [27] was founded by the Ministry of Health in 2001 with the goal of increasing the health status of the population though nutrition via developing recommendations and promoting actions and regulations in this area. The most recent PNNS (2019/2023) includes the goal of reducing UPF consumption by 20% in the next five years, and this concept will be included in the new French Food Guide scheduled to be published in 2021 [51].

## 5. Conclusions

The results of this study show that UPFs make up a sizeable proportion of the diet of French adults, with young individuals showing the highest consumption of these foods. The percentage of UPF consumption presented a positive association with dietary energy density and the dietary contents of total carbohydrates, free sugar, total fat, and saturated fat, as well as with inadequate dietary energy density, and intakes of saturated fat, free sugar, fiber, and potassium. These results support the relevance of public health policies aiming to reduce the access and exposure of the population to UPFs.

## Figures and Tables

**Table 1:** Sociodemographic characteristics of the 2642 adults aged 18–74 years with complete data.

	*n*	%
Sex		
Male	970	36.7
Female	1672	63.3
**Age**		
18–39	900	34.1
40–59	1184	44.8
60+	558	21.1
**Area**		
Rural	611	23.1
Urban	2031	76.9
**Occupation**		
Management/intermediate profession	683	25.9
Self-employed/farmers	102	3.9
Manual workers/employers	802	30.4
Retired	576	21.8
Homemakers, disabled persons, and others	479	18.1
**Education**		
Incomplete high school	379	14.3
Complete high school	1335	50.5
Technical course	376	14.2
University graduate	552	20.9

**Table 2:** Mean daily energy intake according to NOVA food groups. French population aged ≥18 years. Étude Nationale Nutrition Santé (ENNS) 2006 (*n* = 2642).

NOVA Food Groups	kcal	CI * (95%)	% of Total kcal	CI * (95%)
Unprocessed or Minimally Processed Foods and Culinary Ingredients	909.2	890.2; 928.1	44.0	43.3; 44.7
Meat (beef, poultry, pork, and others)	187.8	179.2; 196.4	9.0	8.6; 9.4
Added fat ^1^	108.3	103.6; 113.0	5.3	5.1; 5.5
Fruits	83.9	79.5; 88.2	4.2	4.0; 4.4
Patisseries ^2^	81.7	74.4; 89.0	3.7	3.4; 4.0
Pasta	76.3	70.6; 82.0	3.6	3.4; 3.9
Milk and yogurt	63.1	59.3; 67.0	3.2	3.0; 3.4
Potatoes and other tubers	50.5	47.3; 53.6	2.4	2.3; 2.6
Cereals	43.2	39.2; 47.2	2.1	1.9; 2.3
Table sugar	40.7	38.0; 43.5	1.9	1.8; 2.0
Vegetables	35.7	33.7; 37.7	1.8	1.7; 1.9
Mixed dishes	32.5	29.7; 35.3	1.7	1.5; 1.8
Fish and seafood	33.9	27.8; 40.0	1.6	1.4; 1.9
Eggs	19.2	17.3; 21.1	0.9	0.8; 1.0
Home-made sauces	13.5	11.5; 15.5	0.6	0.5; 0.7
Nuts	13.2	12.1; 14.4	0.7	0.6; 0.7
Legumes	12.0	10.8; 13.2	0.6	0.6; 0.7
Natural fruits juices	7.3	6.1; 8.5	0.4	0.3; 0.4
Coffee and tea	3.7	3.1; 4.2	0.2	0.2; 0.2
Spices	0.5	0.3; 0.7	0.0	0.0; 0.0
Processed foods	538.2	520.8; 555.6	24.9	24.2; 25.5
Bread	289.8	278.5; 301.1	13.5	13.0; 13.9
Cheese	130.4	124.0; 136.8	6.1	5.8; 6.4
Meat products	76.4	68.7; 84.0	3.3	3.0; 3.6
Processed fruits	21.6	19.3; 23.8	1.0	0.9; 1.1
Canned vegetables and legumes	12.4	11.0; 13.8	0.6	0.5; 0.7
Beer and wine	10.1	9.2; 11.0	0.5	0.4; 0.5
Ultra-processed foods	663.3	640.5; 686.1	31.1	30.3; 31.9
Ready-to-eat meals ^3^	168.0	156.7; 179.3	7.9	7.4; 8.4
Confectionery ^4^	115.4	108.6; 122.2	5.4	5.1; 5.7
Cold cuts (charcuterie) and other ultra-processed meats ^5^	92.5	87.5; 97.5	4.4	4.1; 4.6
Bakery products ^6^	56.8	50.6; 63.0	2.6	2.3; 2.8
Sweetened beverages ^7^	50.0	44.3; 55.7	2.3	2.1; 2.6
Dairy products	35.4	32.6; 38.3	1.8	1.6; 1.9
Cookies ^8^	35.9	31.4; 40.3	1.7	1.5; 1.8
Bread	29.6	26.3; 32.9	1.5	1.3; 1.6
Sauces	23.6	21.5; 25.7	1.1	1.0; 1.2
Distilled alcoholic drinks	17.8	12.4; 23.2	0.7	0.5; 0.9
Margarine	12.8	11.1; 14.4	0.6	0.6; 0.7
Breakfast cereals	12.9	10.5; 15.4	0.6	0.5; 0.8
Chips and crackers	6.8	5.3; 8.3	0.3	0.2; 0.3
Cheese ^9^	5.9	5.1; 6.7	0.3	0.3; 0.4
TOTAL	2110.7	2073.9; 2147.4	100.0	-

* Confidence Interval (CI) ^1^ Added fat: includes table fat from animals or vegetables (such as olive oil and butter). ^2^ Patisseries: include homemade sweets and desserts. ^3^ Ready-to-eat meals: include fast-food, noodles, canned or dehydrated soups, pizza, frozen dishes, sandwiches, and other ready-to-eat meals. ^4^ Confectionery: includes sweets (such as chocolate bars, bonbons, gums, lollypops, candy, gummies, ice-cream, torrone, etc.). ^5^ Cold cuts and other ultra-processed meat: include nuggets, sausages, hamburgers, different types of cold cuts/charcuterie (ham, mortadella, and turkey blanquet), and pre-seasoned meat. ^6^ Bakery products: include sweet baked products such as cakes, pies, and sweet breads. ^7^ Sweetened beverages: include soft drinks, artificial juices, and other sweetened beverages; ^8^ Cookies: include every type of ultra-processed cookie. ^9^ Ultra-processed cheese: includes cream-cheese, petit Suisse, and cheese with sweeteners.

**Table 3:** Mean percentage of total energy intake from NOVA food groups according to sociodemographic characteristics. French population aged ≥18 years. ENNS 2006 (*n* = 2642).

	Minimally Processed Food and Culinary Ingredients		Processed Food		Ultra-Processed Food	
	%	CI (95%)	%	CI (95%)	%	CI (95%)
Sex						
Male	41.2	40.2; 42.3	27.4	26.4; 28.4	31.4	30.1; 32.7
Female	46.8	46.0; 47.6	22.3	21.7; 23.0	30.9	30.0; 31.9
**Age**						
18–39	40.5	39.3; 41.7	20.4	19.4; 21.5	39.1	37.8; 40.5
40–59	45.1	44.2; 46.1	26.7	25.9; 27.6	28.1	27.2; 29.0
60+	48.8	47.6; 49.9	29.7	28.6; 30.7	21.6	20.4; 22.8
**Area**						
Rural	43.7	42.5; 45.0	27.3	26.2; 28.5	28.9	27.4; 30.4
Urban	44.1	43.3; 44.9	24.0	23.3; 24.8	31.9	30.9; 32.8
**Occupation**						
Management/intermediate profession	42.8	41.8; 43.9	25.0	24.0; 26.0	32.2	30.9; 33.4
Self-employed/farmers	43.4	40.7; 46.1	28.5	26.3; 30.7	28.1	25.1; 31.2
Manual workers/employees	42.6	41.3; 43.8	24.7	23.7; 25.8	32.7	31.3; 34.2
Retired	48.2	47.0; 49.3	29.5	28.5; 30.6	22.3	21.1; 23.5
Homemakers, disabled persons, and others	43.9	42.2; 45.5	20.2	18.7; 21.8	35.9	34.1; 37.7
**Education**						
Incomplete high school	45.7	44.1; 47.3	27.8	26.4; 29.2	26.5	24.9; 28.1
Complete high school	43.3	42.4; 44.2	23.7	22.9; 24.6	32.9	31.8; 34.1
Technical course	43.9	42.4; 45.4	23.9	22.4; 25.4	32.2	30.3; 34.0
University degree	43.4	42.1; 44.8	24.7	23.4; 25.9	31.9	30.4; 33.4

**Table 4:** Nutrient profile of the whole diet and of three diet fractions. French population ≥18 years. ENNS 2006 (*n* = 2642).

Indicator	Whole Diet	Diet Fraction Made of Unprocessed or Minimally Processed Foods and Culinary Ingredients (*SE*)	Diet Fraction Made of Processed Foods (*SE*)	Diet Fraction Made of Ultra-Processed Foods (*SE*)
Total energy (kcal/day)	2110.7	909.2 (9.7)	538.2 (8.9)	663.3 (11.6)
Energy density (kcal/g)	1.5	1.2 (0.0)	2.0 (0.0)	2.0 (0.0)
Protein (% of total energy)	17.5	22.0 (0.2)	15.9 (0.1)	12.7 (0.1)
Carbohydrates (% of total energy)	41.4	37.3 (0.3)	47.1 (0.4)	42.6 (0.4)
Total sugar (% of total energy)	19.0	21.3 (0.3)	7.3 (0.2)	25.5 (0.4)
Free sugar (% of total energy)	11.7	8.1 (0.2)	6.0 (0.2)	21.7 (0.4)
Total fat (% of total energy)	37.6	40.5 (0.3)	26.5 (0.4)	42.1 (0.3)
Saturated fat (% of total energy)	15.7	15.6 (0.1)	14.3 (0.2)	17.2 (0.2)
Fiber (g/1000 kcal/day)	8.9	11.2 (0.1)	7.4 (0.1)	7.1 (0.1)
Potassium (mg/1000 kcal/day)	1491.8	2130.6 (17.1)	726.8 (8.2)	1304.9 (16.3)
Sodium (mg/1000 kcal/day)	1485.3	1254.6 (12.7)	2022.5 (19.1)	1526.3 (19.8)

**Table 5:** Nutrient intake indicators of the overall population and according to quintiles of ultra-processed food consumption. French population aged ≥18 years old. ENNS 2006 (*n* = 2642).

Nutritional Indicators ^a^	Overall Population		Quintiles of Ultra-Processed Food Consumption (% of Total Energy Intake) ^b^					Regression Coefficient		
	Mean	Standard Deviation (*SD*)	1st Quintile (lowest)	2nd Quintile	3rd Quintile	4th Quintile	5th Quintile (Highest)	Adjusted ^c^	Adjusted and Standardized ^c^	*P* *
Total energy (kcal)	2110.7	18.7	2069.2	2140.8	2109.6	2139.7	2093.5	4.52	0.01	0.772
Energy density (kcal/g)	1.5	0.0	1.4	1.4	1.5	1.5	1.6	0.05	0.18	0.000
Protein (% of total energy)	17.5	0.1	18.8	18.0	17.7	17.0	16.0	−0,66	−0.26	0.000
Carbohydrates (% of total energy)	41.4	0.2	40.5	41.1	41.0	42.4	41.9	0.39	0.08	0.006
Free sugar (% of total energy)	11.7	0.2	8.7	10.9	11.8	13.0	14.1	1.27	0.31	0.000
Total fat (% of total energy)	37.6	0.2	36.8	36.9	37.7	37.6	38.8	0.46	0.11	0.000
Saturated fat (% of total energy)	15.7	0.1	15.3	15.6	15.9	15.8	15.9	0.15	0.06	0.035
Fiber density (g/1000 kcal)	8.9	0.1	9.5	9.1	8.8	8.8	8.1	−0.31	−0.14	0.000
Potassium (mg/1000 kcal)	1491.8	9.5	1550.4	1508.8	1491.5	1490.3	1424.1	−26.99	−0.10	0.000
Sodium density (g/1000 kcal)	1485.3	9.2	1510.5	1504.9	1471.7	1485.5	1456.5	−12.94	−0.05	0.059

^a^ All values refer to means; ^b^ Average of UPF: first quintile (*n* 648): 12.8% UPF (min: 0.1%/max: 18.3%); second quintile (*n* 613): 22.0% UFP (min: 18.3%/max: 25.5%); third quintile (*n* 633): 29.0% UPF (min: 25.6%/max: 32.7%); fourth quintile (*n* 596): 36.2% UPF (min: 32.7%/max: 42.1%); fifth quintile (*n* 487): 51.5% UPF (min: 42.1%/max: 88.6%). ^c^ Adjusted for total energy intake and sociodemographic characteristics (age, sex, area, region, occupation, and education level); * Tests of linear trend were performed by treating quintiles of the dietary share of ultra-processed food as an ordinal variable; SD, standard deviation.

**Table 6:** Prevalence of inadequate nutrient intake across overall population and according to quintiles of ultra-processed food consumption. French population aged ≥18 years old. ENNS 2006 (*n* = 2642).

Quintiles of Ultra-Processed Food Consumption (% of Total Energy Intake) ^a^	Energy Density < 1.25 kcal/g or > 1.45 kcal/g ^b^		Energy Density < 1.25 kcal/g ^b^		Energy Density > 1.45 kcal/g ^b^		Saturated Fat > 12% of Total Energy Intake ^c^		Free Sugar > 100 g/day ^c^		Fiber ≤ 30 g/day ^c^		Potassium Density < 1755 mg/1000 kcal ^d^		Sodium Density > 1 g/1000 kcal ^d^	
	%	OR ^e^	%	OR ^e^	%	OR ^e^	%	OR ^e^	%	OR ^e^	%	OR ^e^	%	OR ^e^	%	OR ^e^
1st quintile (lowest)	72.3	1.0	44.4	1.0	27.9	1.0	78.8	1.0	3.1	1.0 *	88.1	1.0 *	64.7	1.0	97.1	1.0
2nd quintile	73.7	0.9	35.8	0.8	37.8	1.2	85.9	1.3	6.2	2.6 *	92.7	2.2 *	73.9	1.5 *	95.4	0.7
3rd quintile	74.8	1.0	30.8	0.7 *	44.0	1.6 *	87.7	1.7 *	7.9	3.1 *	94.0	2.1 *	76.2	1.5 *	96.8	1.1
4th quintile	75.0	0.9	22.0	0.7 *	52.9	1.4	89.2	1.5	13.3	4.0 *	95.4	2.1 *	80.3	1.3	94.3	0.5
5th quintile (highest)	87.4	2.0 *^,†^	9.2	0.3 *^,†^	78.2	3.9 *^,†^	91.2	2.5 *^,†^	23.2	6.7 *^,†^	98.0	5.9 *^,†^	90.3	3.5 *^,†^	88.7	0.4 *^,†^
Total	76.2	-	29.3	-	46.9	-	86.3	-	10.2	-	93.5	-	76.5	-	94.7	-

^a^ Average of UPF: first quintile (*n* 648): 12.8% UPF (min: 0.1%/max: 18.3%); second quintile (*n* 613): 22.0% UFP (min: 18.3%/max: 25.5%); third quintile (*n* 633): 29.0% UPF (min: 25.6%/max: 32.7%); fourth quintile (*n* 596): 36.2% UPF (min: 32.7%/max: 42.1%); fifth quintile (*n* 487): 51.5% UPF (min: 42.1%/max: 88.6%). ^b^ World Cancer Research Foundation (WCRF). Energy density: finding the balance for cancer prevention. London: World Cancer Research Fund; 2009. ^c^ Agence nationale de sécurité sanitaire de l’alimentation, de l’environnement et du travail (ANSES). Actualisation des repères du PNNS: élaboration des références nutritionnelles. ANSES, 2016. ^d^ World Health Organization (WHO), World Health Organization issues new guidance on dietary salt and potassium, Geneva; 2013. ^e^ ORs (odds ratios) were adjusted for total energy intake and sociodemographic characteristics (age, sex, area, region, occupation, and education level); * Statistically significant *p* < 0,05; ^†^ Tests of linear trend were performed by treating quintiles of the dietary share of ultra-processed food as an ordinal variable (*p* < 0.05).

## Data Availability

The data that support the findings of this study are available from Santé Publique France but restrictions apply to the availability of these data, which were used under license for the current study, and so are not publicly available. Data are however available from the authors upon reasonable request and with permission from Santé Publique France.

## Supplementary Materials

The following are available online at https://www.mdpi.com/2072-6643/13/2/682/s1: Table S1: Sociodemographic characteristics of the 2642 adults aged 18–74 years with complete data.
