# Supplementary material for: Consumption of Ultra-Processed Food and Its Association with Sociodemographic Characteristics and Diet Quality in a Representative Sample of French Adults
Source: Nutrients. 2021 Feb 20;13(2):682. doi: 10.3390/nu13020682 (PMC7924346; doi:10.3390/nu13020682)
Supplement: Supplementary file 1 [file nutrients-13-00682-s001.pdf]

Supplementary Material

Table S1. Mean daily grams intake according to NOVA food groups. French population aged ≥ 18 years. ENNS 2006 (*n* = 2642).

NOVA Food Groups	Grams	CI (95%)		% of Total Grams	CI (95%)	
Unprocessed or minimally processed foods and culinary ingredients	1146.1	1121.8	1170.4	59.9	59	60.7
Meat (beef, poultry, pork, and others)	99.4	95.1	103.8	5.4	5.2	5.6
Added fat [1]	16.9	16.1	17.7	0.9	0.9	1
Fruits	168.7	160	177.4	8.6	8.2	9
Patisseries [2]	34.4	31.3	37.4	1.8	1.7	1.9
Pasta	50.5	47.2	53.9	2.8	2.6	3
Milk and yogurt	122.2	114.5	130	6.5	6.1	6.9
Potatoes and other tubers	49.6	46.6	52.7	2.6	2.4	2.8
Cereals	30.7	28	33.4	1.7	1.5	1.9
Table sugar	10.4	9.7	11.1	0.5	0.5	0.6
Vegetables	157.6	150.4	164.9	8.2	7.9	8.6
Mixed dishes	28.3	25.9	30.6	1.5	1.4	1.7
Fish and seafood	18.7	15.8	21.5	1.1	0.9	1.3
Eggs	11.8	10.6	12.9	0.6	0.6	0.7
Home-made sauces	2.6	2.2	3	0.1	0.1	0.2
Nuts	3.8	3.4	4.2	0.2	0.2	0.2
Legumes	24.7	22.6	26.7	1.4	1.2	1.5
Natural fruits juices	17.8	15	20.6	0.9	0.8	1.1
Coffee and tea	294.2	279.7	308.8	14.6	14	15.3
Spices	3.2	1.8	4.6	0.2	0.1	0.2
Processed foods	317.4	301.6	333.2	16	15.4	16.6
Bread	103.6	99.6	107.7	5.5	5.3	5.7
Cheese	46.8	44.5	49.2	2.5	2.4	2.6
Meat products	122.1	107.6	136.5	5.6	5.1	6.2
Processed fruits	9.9	9	10.8	0.5	0.5	0.6
Canned vegetables and legumes	20.4	18	22.8	1.1	1	1.2
Beer and wine	14.6	13.2	15.9	0.8	0.7	0.9

Ultra-processed foods	451.4	433	469.7	24.1	23.3	25
Ready-to-eat meals [3]	124.8	118.3	131.3	6.7	6.4	7.1
Confectionery [4]	48.2	45.3	51.1	2.6	2.5	2.8
Cold cuts (charcuterie) and other ultra-processed meats [5]	39.3	37.2	41.4	2.2	2	2.3
Bakery products [6]	16.4	14.7	18.2	0.9	0.8	1
Sweetened beverages [7]	127.6	113.2	141.9	6.5	5.9	7.2
Dairy products	45.9	42.4	49.5	2.5	2.3	2.7
Cookies [8]	8.1	7.1	9.1	0.4	0.4	0.5
Bread	9.6	8.4	10.8	0.6	0.5	0.6
Sauces	9.9	9.1	10.7	0.5	0.5	0.6
Distilled alcoholic drinks	10.3	7.6	13.1	0.5	0.4	0.6
Margarine	2.4	2.1	2.7	0.1	0.1	0.1
Breakfast cereals	3.5	2.8	4.1	0.2	0.2	0.2
Chips and crackers	1.4	1.1	1.7	0.1	0.1	0.1
Cheese [9]	3.6	3	4.1	0.2	0.2	0.2
TOTAL	1914.9	1881.9	1947.8	100		

1: Added fat: includes table fat from animal or vegetable (such as oil, olive, and butter). 2: Patisseries: include homemade sweets and desserts. 3: Ready-to-eat meals: include fast-food, noodles, canned or dehydrated soups, pizza, frozen dishes, sandwiches, and other ready-to-eat meals. 4: Confectionery: includes sweets (such as chocolate bars, bonbons, gums, lollypop, candy, gummies, ice-cream, torrone, etc.). 5: Cold cuts and other ultra-processed meat: include nuggets, sausages, hamburgers, different types of cold cuts/charcuterie (ham, mortadella, and turkey blanquet), and pre-seasoned meat. 6: Bakery products: include sweet baked products such as cakes, pies, and sweet breads. 7: Sweetened beverages: include soft drinks, artificial juices, and other sweetened beverages; 8: Cookies: include every type of ultra-processed cookie. 9: Ultra-processed cheese: includes cream-chesse, petit suisse and cheese with sweeteners.

Table S2. Nutrient intake indicators of the overall population and according quintiles of ultra-processed food consumption. French population aged ≥ 18 years old. ENNS 2006 (*n* = 2642).

Nutritional Indicators[a]	Overall Population		Quintiles of Ultra-Processed Foods Consumption (% of Total Grams Intake) [b]					Regression Coefficient		
	Mean	SD	1st Quintile (Lowest)	2nd Quintile	3rd Quintile	4th Quintile	5th Quintile (Highest)	Adjusted [c]	Adjusted and Standardized [c]	*p**
Total energy (kcal)	2110.7	18.7	2025.6	2128.0	2119.9	2109.3	2171.0	27,06	0.06	0.015
Energy density (kcal/g)	1.5	0.0	1.4	1.5	1.5	1.5	1.6	0.05	0.19	0.000
Protein (% of total energy)	17.5	0.1	18.2	18.0	17.2	17.4	16.5	−0.41	−0.16	0.000
Carbohydrates (% of total energy)	41.4	0.2	40.6	39.8	41.9	42.1	42.6	0.65	0.13	0.000
Free sugar (% of total energy)	11.7	0.2	9.2	10.2	11.6	12.4	15.3	1.40	0.35	0.000
Total fat (% of total energy)	37.6	0.2	36.9	37.8	37.1	37.8	38.5	0.31	0.08	0.008
Saturated fat (% of total energy)	15.7	0.1	15.3	15.6	15.6	16.2	15.9	0.18	0.07	0.012
Fiber density (g/1000 kcal)	8.9	0.1	9.4	9.0	9.0	8.6	8.1	−0.29	−0.13	0.000
Potassium (mg/1000kcal)	1491.8	9.5	1557.4	1503.9	1502.0	1475.8	1419.5	−29.70	−0.11	0.000
Sodium density (g/1.000 kcal)	1485.3	9.2	1487.6	1485.6	1500.8	1493.0	1459.2	−3.97	0.02	0.539

a: All values refer to means; b: Average of UPF: 1st quintile (*n* 624): 8.2% UPF (min: 0.2%/max: 12.3%); 2nd quintile (*n* 588): 15.4% UFP (min: 12.3%/max: 18.3%); 3rd quintile (*n* 548): 21.3% UPF (min: 18.3%/max: 24.7%); 4th quintile (*n* 510): 29.0% UPF (min: 24.7%/max: 34.6%); 5th quintile (*n* 372): 45.1% UPF (min: 34.6%/max: 85.5%). c: Adjusted for total energy intake and sociodemographic characteristics (age, sex, area, region, occupation and education level); * Tests of linear trend were performed by treating quintiles of the dietary share of ultra-processed food as an ordinal variable; SD, standard deviation.

Table S3. Prevalence of inadequate nutrient intake across overall population and according quintiles of ultra-processed food consumption. French population aged ≥ 18 years old. ENNS 2006 (n = 2642).

Quintiles of Ultra-Processed Foods Consumption (% of Total Grams Intake)[a]	Energy Density < 1.25 kcal/g or > 1.45 kcal/g[b]		Energy Density < 1.25 kcal/g[b]		Energy Density > 1.45 kcal/g[b]		Saturated Fat > 12% of Total Energy Intake[c]		Free Sugar > 100 g/day[c]		Fiber ≤ 30 g/day[c]		Potassium Density < 1755 mg/1000 kcal[d]		Sodium Density > 1 g/1000 kcal[d]	
	%	PR[e]	%	PR[e]	%	PR[e]	%	PR[e]	%	PR[e]	%	PR[e]	%	PR[e]	%	PR[e]
**1st quintile (lowest)**	73.4	1.0	42.1	1.0	31.3	1.0	80.4	1.0	2.9	1.0	90.5	1.0	65.4	1.0	95.7	1.0
**2nd quintile**	70.4	0.9	33.2	0.9	37.2	1.1	86.4	1.4	6.1	1.9	92.2	1.9*	75.2	1.2	96.4	1.3
**3rd quintile**	74.5	0.9	29.6	0.7*	44.9	1.3	87.0	1.3	8.2	1.7	92.3	1.3	75.5	1.3	95.6	0.9
**4th quintile**	79.8	1.2	22.2	0.6*	57.6	1.7*	90.4	2.2*	13.7	3.5*	96.5	3.0*	83.9	1.8*	93.7	0.8
**5th quintile (highest)**	87.6	1.9*†	11.3	0.4*†	76.3	2.8*†	89.5	2.2*†	26.9	5.8*†	97.8	3.7*†	88.4	2.5*†	90.6	0.7
**Total**	76.2	-	29.3	-	46.9	-	86.3	-	10.2	-	93.5	-	76.5	-	94.7	-

a: Average of UPF: 1st quintile (n 624): 8.2% UPF (min: 0.2%/max: 12.3%); 2nd quintile (n 588): 15.4% UFP (min: 12.3%/max: 18.3%); 3rd quintile (n 548): 21.3% UPF (min: 18.3%/max: 24.7%); 4th quintile (n 510): 29.0% UPF (min: 24.7%/max: 34.6%); 5th quintile (n 372): 45.1% UPF (min: 34.6%/max: 85.5%). b: World Cancer Research Foundation (WCRF). Energy density: finding the balance for cancer prevention. London: World Cancer Research Fund; 2009. c: Agence nationale de sécurité sanitaire de l'alimentation, de l'environnement et du travail (ANSES). Actualisation des repères du PNNS: élaboration des références nutritionnelles. ANSES, 2016. d: World Health Organization (WHO), World Health Organization issues new guidance on dietary salt and potassium, Geneva; 2013. e: PR (Prevalence Ratio) were adjusted for total energy intake and sociodemographic characteristics (age, sex, area, region, occupation and education level); * Statistically significant $p < 0,05$; † Tests of linear trend were performed by treating quintiles of the dietary share of ultra-processed food as an ordinal variable ($p < 0.05$).
